# Increased DJ-1 in Urine Exosome of Korean Males with Parkinson's Disease

**DOI:** 10.1155/2014/704678

**Published:** 2014-11-13

**Authors:** Dong Hwan Ho, Sanghak Yi, Hyemyung Seo, Ilhong Son, Wongi Seol

**Affiliations:** ^1^InAm Neuroscience Research Center, Sanbon Medical Center, College of Medicine, Wonkwang University, Sanbon-dong, Gunpo-si, Gyeonggi-do, Republic of Korea; ^2^Department of Molecular and Life Sciences, Hanyang University, Ansan-si, Gyeonggi-do, Republic of Korea; ^3^Department of Neurology, Sanbon Medical Center, College of Medicine, Wonkwang University, Sanbon-dong, Gunpo-si, Gyeonggi-do, Republic of Korea

## Abstract

Parkinson's disease (PD) is a difficult disease to diagnose although it is the second most common neurodegenerative disease. Recent studies show that exosome isolated from urine contains LRRK2 or DJ-1, proteins whose mutations cause PD. To investigate a potential use for urine exosomes as a tool for PD diagnosis, we compared levels of LRRK2, *α*-synuclein, and DJ-1 in urine exosomes isolated from Korean PD patients and non-PD controls. LRRK2 and DJ-1, but not *α*-synuclein, were detected in the urine exosome samples, as reported previously. We initially could not detect any significant difference in these protein levels between the patient and the control groups. However, when age, disease duration, L-dopa daily dose, and gender were considered as analytical parameters, LRRK2 and DJ-1 protein levels showed clear gender-dependent differences. In addition, DJ-1 level was significantly higher (1.7-fold) in male patients with PD than that in male non-PD controls and increased in an age-dependent manner in male patients with PD. Our observation might provide a clue to lead to a novel biomarker for PD diagnosis, at least in males.

## 1. Introduction

Parkinson's disease (PD) is the most common motor neurodegenerative disease of the elderly. The major symptoms of PD are bradykinesia, resting tremor, rigidity, and unstable posture and pathological hallmarks of PD are selective degeneration of dopaminergic (DA) neurons in the substantia nigra pars compacta and formation of Lewy bodies whose main component is *α*-synuclein in surviving neurons. However, many reports suggested that the pathology of PD also includes other areas of the central nervous system, the autonomic nervous system, and the enteric nervous system [1, 2]. Recently, nonmotor symptoms of PD were also emphasized in addition to motor symptoms [3]. Although PD cases are increasing worldwide as the elderly population increases, a clinical diagnosis in early stages of the disease is difficult because of a lack of suitable disease biomarkers and overt clinical symptoms.

Exosomes are microvesicles secreted from cells to biofluids such as blood, urine, and cell culture medium. Purified exosomes reportedly contain specific proteins, microRNAs, and mRNAs that could be a signature of the originating cells [4]. Because proteomic analysis of urine exosomes also displays characteristics of renal cells from which the exosomes originated, the possibility of using urine exosomes as a tool for low-cost screening of renal diseases has been suggested [5–7].

From researches on familial PD patients, several genes such as DJ-1, LRRK2, and *α*-synuclein [8, 9] have been reported as PD-causative genes. Proteomic analyses of urine exosomes have reported the presence of PD causative gene products such as LRRK2 and DJ-1 [10, 11]. However, *α*-synuclein, another PD causative gene product that is being intensively studied for a putative PD biomarker [12, 13], has not been detected [10, 11] although its isoform, *γ*-synuclein, has been detected in urine exosomes [11]. One study reported that LRRK2, with its interacting partner 14-3-3, are present in urine exosomes purified from patients with PD, but no significant difference in LRRK2 level was detected between PD and control groups [10].

In this study, we isolated urine exosomes from PD and non-PD control groups among the Korean population using a filtration method and analyzed the levels of LRRK2 and DJ-1 by Western blot. We included *α*-synuclein in our analysis because its expression level in the brain is strongly related to PD pathogenesis [14–16]. Our results show that the amount of DJ-1 increased in urine exosome of male patients with PD, but not in female patients with PD.

## 2. Material and Methods

### 2.1. Clinical Samples

This study was approved by the Institutional Review Board of Sanbon Medical Center, Wonkwang University (IRB2013-24). Urine specimens from 27 patients with PD and 27 age- and gender-matched non-PD controls (Table 1) were originally collected at the Department of Neurology, Sanbon Medical Center, and stored at −80°C for 1–3 months until use. All PD patients were diagnosed by a neurologist based on the UK Brain Bank criteria [17]. Non-PD controls were collected from patients hospitalized at Sanbon Medical Center and their caregivers who had no clear PD symptoms as assessed by a neurologist. All donors signed a consent form. Information on patients' PD-related criteria such as Hoehn/Yahr (HY) or Unified Parkinson's Disease Rating Scale (UPDRS) scores was unavailable, but their disease duration times since the first diagnosis and L-dopa dosages were available and used for analysis.

During analysis, we performed a urinalysis of samples and excluded seven proteinuria samples that contained excess proteins in the urine from the statistical analysis [18].

### 2.2. Exosome Isolation

Ultracentrifugation and microfiltration are the two most frequently used methods to prepare exosome [6, 19, 20]. One report compared both methods and concluded that both procedures yielded equivalent enrichment of the exosome proteome [21]. Because microfiltration is simple and suitable for routine clinical laboratory applications, we chose a microfiltration method using Vivaspin-20 with a MWCO of 100,000 to prepare the exosomes [6, 19]. Urine samples were slowly thawed in ice overnight and vortexed extensively for 90 sec at maximum speed. Protease inhibitors (0.5 mL of 100 mM NaN_3_, 0.75 mL of 10 mM PMSF, and 24 *μ*L of 1 mM leupeptin) were sequentially added to 15 mL of urine and mixed [6]. The samples were centrifuged at 17,000 ×g for 15 min at 4°C, and the supernatant was filtered using a PBS-prewashed Vivaspin 20 (100 kDa MWCO, Sartorius Stedim, Gottingen, Germany) at 3,000 ×g for 30 min at room temperature. The remaining sample in a filter cup, the retentate (R), was mixed with the same volume of 2x solubilizing buffer (2x Laemmli buffer with 400 mM dithiothreitol). Proteins that adhered to the filter membrane were extensively washed with 250 *μ*L of 1x solubilizing buffer (1x Laemmli buffer with 200 mM dithiothreitol) and designated as the heated SDS-containing wash (HSW). Both solubilizing buffers were prewarmed to 90°C before use.

### 2.3. Western Blot Analysis

Fifteen *μ*L of the HSW from each sample was loaded on four gradient SDS-protein gels (Bio-Rad, 4–15%; Hercules, CA, USA) so that a gender- and age-matched patient/control pair was loaded side by side. In total, we analyzed 27 PD samples and 27 gender- and age-matched non-PD samples (14 male and 13 female pairs). The neuroblastoma SH-SY5Y cells were harvested after differentiation with 10 *μ*M retinoic acid for 6 days and lysed with lysis buffer (1% triton X-100 in PBS, 1x protease inhibitor cocktail (Genedepot, Barker, TX, USA)). The SH-SY5Y cell lysates were used as a positive control for the western blot analysis. All samples in the gels were processed for Western blot analysis under the same conditions for each antibody: LRRK2 (MJFF2 Abcam, Cambridge, MA, USA; ab133474, 1 : 1000), *α*-synuclein (Cell Signalling Technology (CST), Danvers, MA, USA; #2642, 1 : 800), DJ-1 (CST, #2134, 1 : 1000), and TSG101 (Abcam, ab83, 1 : 500). Specific bands of the four separate blots were detected with an enhanced chemiluminescence reagent (WBLUR0500, Millipore, Milford, MA, USA) using the Microchemi 4.2 device (DNR Bioimaging Systems, Jerusalem, Israel) under the same conditions and their densities were analyzed with the Multi-Gauge V 3.0 program (Fuji photo Film, Tokyo, Japan).

### 2.4. Data Analysis

Band density was divided by the density of its corresponding exosome loading control, TSG101, and their values were grouped by gender, disease status, and age. The mean of each group was calculated and compared to its corresponding value. The statistical analysis was carried out with the Prism5 program (GraphPad Software, La Jolla, CA, USA). Detailed information on the statistical analysis is given in each figure legend.

## 3. Results 

### 3.1. The HSW Sample Contains More Protein Than the R Sample

**Figure 1:**
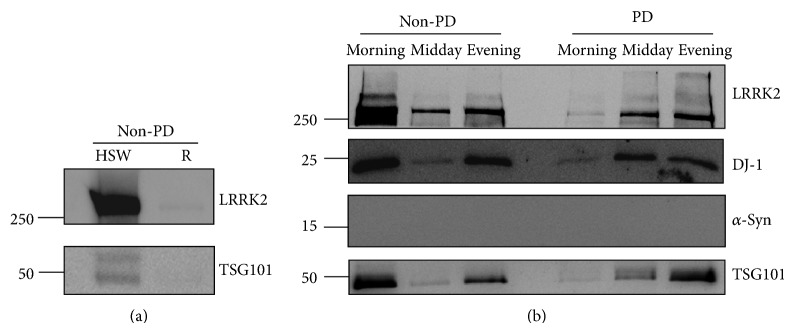
Western blot results of urine exosome (a). A comparison of heated SDS-containing wash (HSW) and retentate (R) samples isolated from urine of a female non-PD control. The same volume of each exosome sample was loaded on a SDS-protein gel and analyzed with LRRK2 and TSG101 antibodies. (b) Urine was sampled at different times of the same day from one female with non-PD and one female with PD. LRRK2, DJ-1, *α*-synuclein, and TSG101 were analyzed in the HSW samples. Approximate sampling times are indicated.

We investigated whether the levels of proteins encoded from PD-causative genes such as LRRK2, *α*-synuclein, and DJ-1 were different in urine exosomes purified from Korean PD and non-PD control groups. We used a filtration protocol reported previously [19], which generated R and HSW samples from each urine sample. Analysis of both samples by Western blot indicated that the HSW contained more protein than that in the R samples as reported previously [19, (Figure 1(a))]. Therefore, we used the HSW for further analysis. Before extensive analysis, we investigated whether urine sampling time may have affected the pattern of each protein, as first-morning urine contains more proteins. Our analysis suggested that sampling time did not affect relative protein amount in the exosomes after normalization to TSG101, as reported previously [6], although their absolute amount might vary depending on sampling time (Figure 1(b)). Therefore, we collected urine sample when patients visited our clinic and signed the consent form regardless of time. This could be advantageous for clinical application because a specific sampling time was not required.

**Figure 2:**
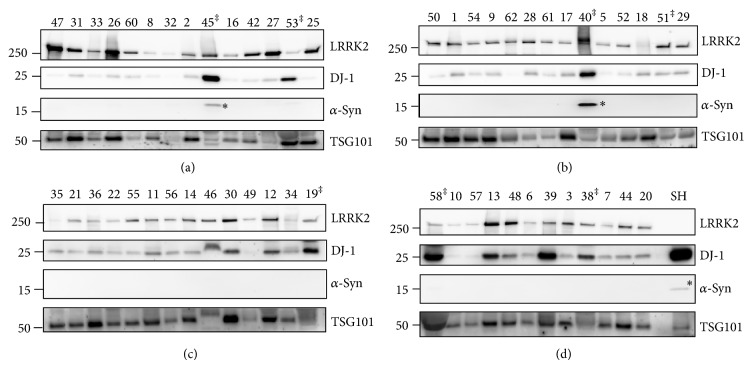
Western blot results of all exosome samples. Exosome samples isolated from PD and non-PD urine samples were loaded as age- and gender-matched PD and non-PD sample pairs side by side on SDS-protein gels and analyzed by Western blotting with LRRK2, DJ-1, *α*-synuclein (*α*-syn), and TSG101 antibodies under the same conditions at the same time. The membrane was cut to the proper size and each membrane was blotted with the indicated antibody. Molecular weight markers are indicated on the left side of the blots. SH and SH-SY5Y cell lysates were used as a positive control; ^‡^ samples excluded from analysis because the urinalysis showed presence of protein; ^*^ monomeric form of *α*-synuclein. Numbers ≤ 31 indicate PD and numbers ≥ 32 indicate non-PD control samples. The male samples are shown in gels (a) and (b), and the female samples are shown in gels (c) and (d).

The HSW samples from the PD and gender- and age-matched control pairs were loaded on SDS-protein gels side by side and analyzed by Western blot with LRRK2, DJ-1, *α*-synuclein, and TSG101 antibodies (Figure 2). During analysis, we noticed that some urine samples contained proteins that could interfere with the purification or analysis steps [22] and these proteins could indicate renal disease that could affect the exosome proteomic pattern [23]. Therefore, we performed a urine dipstick analysis and found that one PD (sample no. 19) and six non-PD samples (samples numbers 38, 40, 45, 51, 53, and 38) contained protein. Therefore, we excluded these samples from analysis, resulting in a decrease of sample size to 26 PD (14 males and 12 females) and 21 non-PD samples (10 males and 11 females). This exclusion caused a slight age difference of approximately 2 years between the PD and the non-PD control groups. In addition, these excluded samples contained more extensive degradation of the TSG101 (Figure 2 lanes C-19, D-58, and D-38) and LRRK2 proteins (Figure 2 lanes A-45 and B-40), supporting our exclusion of proteinuria samples from the analysis.

### 3.2. *α*-Synuclein Was Not Detected in Urine Exosomes

The Western blot result with the *α*-synuclein antibody showed no specific *α*-synuclein monomeric protein band at 15 Kda in our samples, although a specific band was detected in the positive control, the lysates of SH-SY5Y cells, and some proteinuria samples that were later excluded from analysis (Figure 2 lanes A-45, B-40, and D-SH). Therefore, we confirmed the results of previous proteomic analyses of urine exosome proteins in which no *α*-synuclein was detected [10, 11].

### 3.3. DJ-1 Level Was Higher in Male Patients with PD Than That in Male Non-PD Controls

The TSG101 protein is an exosome marker and was used as an internal loading control to normalize the amount of exosome protein loaded. Band densities of LRRK2 and DJ-1 were measured and divided by the density of the corresponding TSG101, and each band density of the PD group was compared to that of the control group.

**Figure 3:**
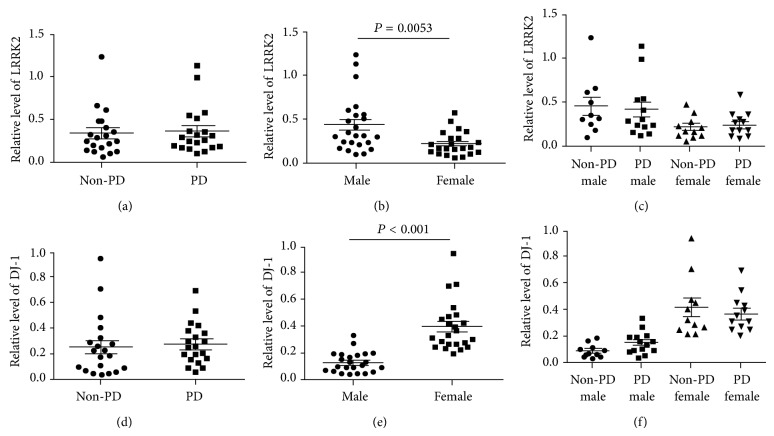
An increase in the DJ-1 level in male patients with PD. ((a), (d)) Analysis of LRRK2 (a) and DJ-1 (d) density by disease status. The density of the indicated protein band was measured and divided by the density of the corresponding TSG101 band. *N* = 21 and 26 in non-PD control and PD, respectively; ((b), (e)) analysis of LRRK2 (b) and DJ-1 (e) density by gender. *n* = 24 and 23 in males and females, respectively. The two-tailed *P* values were calculated by the unpaired *t*-test. ((c), (f)) Analysis of LRRK2 (c) and DJ-1 (f) density by gender and disease status. *N* = 10 in non-PD male; *n* = 14 in PD male; *n* = 11 in non-PD females; and *n* = 12 in PD females.

**Figure 4:**
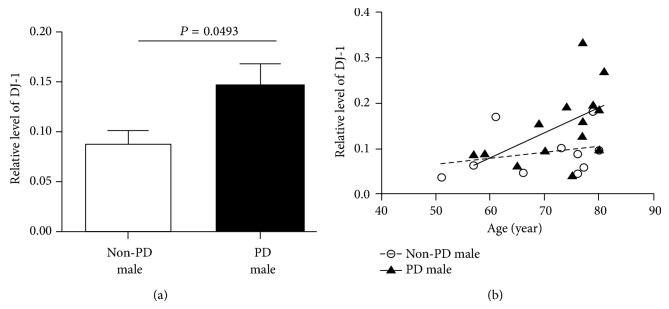
Further analysis of DJ-1 levels in males. (a) A quantitative comparison of LRRK2 and DJ-1 levels in the non-PD control and PD males. The two-tailed *P* values were calculated by the unpaired *t*-test. (b) Analysis of DJ-1 levels in PD and non-PD males with age. The correlation coefficients of PD and non-PD were 0.28 and 0.07, respectively, suggesting almost no correlation between age and DJ-1 level in non-PD males.

**Figure 5:**
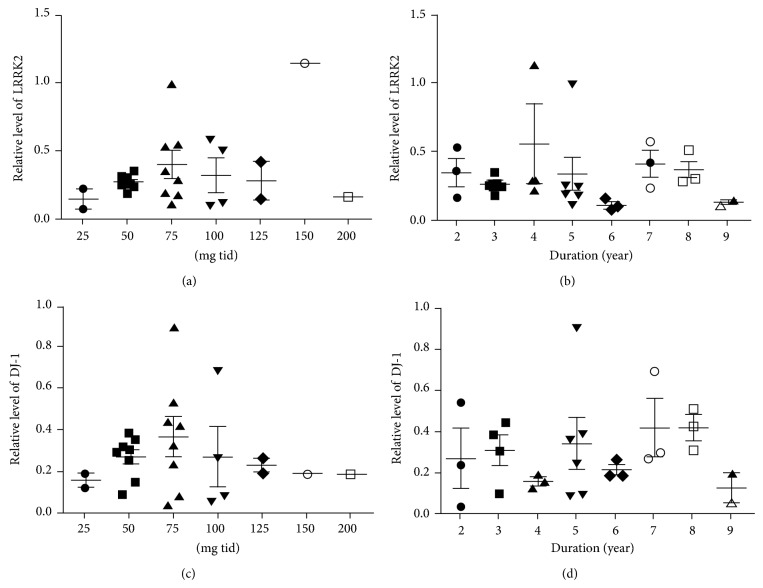
Analysis of levels of LRRK2 ((a), (b)) and DJ-1 ((c), (d)) by L-levodopa dose ((a), (c) mg tid means mg three times per day) and disease duration ((b), (d)).

The relative proteins levels of LRRK2 and DJ-1 were compared between the PD and the non-PD groups, but no differences were detected (Figures 3(a) and 3(d)). Previous studies have reported that *α*-synuclein levels in plasma, cerebrospinal fluid (CSF), and DA neurons are different between males and females [24–26] although there are also reports showing no gender-dependent difference in *α*-synuclein levels in CSF [13, 27]. To include gender as an analysis parameter, we subdivided each group by gender (Figures 3(b) and 3(e)). The result clearly indicated that levels of the LRRK2 and DJ-1 proteins were significantly different between males and females (LRRK2: 0.44 ± 0.063 in males versus 0.24 ± 0.027 in females; *P* = 0.0053 and DJ-1: 0.12 ± 0.016 in males versus 0.39 ± 0.039 in females; with *P* < 0.0001). Levels of LRRK2 were lower in females, whereas the DJ-1 level was the opposite. When disease status was further included in this analysis, only DJ-1 in males with PD (*n* = 14) was 1.7-fold higher (PD: 0.15 ± 0.022 versus non-PD: 0.087 ± 0.016; *P* = 0.0493, Figure 4(a)) than that in non-PD males (*n* = 10). In contrast, no significant difference was observed in DJ-1 level in females or LRRK2 in males or females when both gender and disease status were considered in the analysis (Figures 3(c) and 3(f)). Because age, the most important risk factor for PD, often affects the proteomic pattern in patients with PD [27], we further analyzed DJ-1 level with age in PD and non-PD males. Interestingly, the result showed positive relationship between DJ-1 level and age in PD with a *P* value [*P* = 0.0515, Figure 4(b)], a value close to a statistical significant *P* value (*P* ≤ 0.05). However, the DJ-1 levels in non-PD males were not much different with increased age (*P* = 0.4590, Figure 4(b)). We also analyzed LRRK2 level with age, but no significant difference in LRRK2 level with age was observed (data not shown). PD-related criteria such as UPDRS or HY scores of the patients enrolled in our study were unavailable. Instead, we analyzed both LRRK2 and DJ-1 levels with either daily L-dopa dose or disease duration. Neither analysis detected any significant increase (Figure 5).

## 4. Discussion

PD is the second most common neurodegenerative disease. However, it is difficult to diagnose, as PD symptoms are not apparent until almost 50% of the DA neurons have died [28]. Therefore, developing a biomarker for early detection of PD is an important and urgent task. Urine exosome is a good source to detect noninvasive biomarkers for various diseases because their contents are a molecular fingerprint of cells from which the exosomes were derived and urine is an easily accessible biofluid [29]. In this study, we wanted to test the potential of urine exosomes as a PD biomarker. CSF has been utilized as a source to develop PD biomarkers because PD is caused by death of DA neurons in the midbrain. However, using urine as such a source is significant because most PD causative genes are constitutively expressed, and DJ-1 and LRRK2 are expressed in kidney [30–32]. Besides, PD is gradually recognized as a systemic disorder affecting the autonomic nervous system and the enteric nervous system in addition to the central nervous system, [1, 2].

Several urine exosome proteomic studies have been conducted [10, 11, 18]. Among them, two studies reported the presence of LRRK2 and DJ-1 [10, 11], whereas another one reported the presence of only DJ-1 [18]. No study has reported other PD causative gene products such as Parkin, Pink1, or *α*-synuclein. Therefore, we decided to detect LRRK2 and DJ-1 first.

We also detected LRRK2 and DJ-1 in urine exosomes and showed that only DJ-1 level was significantly different between PD and non-PD males, but not in females. LRRK2 levels were not different between PD and non-PD groups, confirming a previous report (Figures 3 and 4 and [10]). However, our study showed a pattern where DJ-1 level increased with increasing age in PD male samples (Figure 4(b)). Therefore, our results suggest that DJ-1 level in male urine exosomes could be utilized as a PD diagnosis biomarker. However, the sample size in our study was too small to draw a meaningful conclusion and requires an extensive study with more samples to verify our results.

Several studies reported utilizing *α*-synuclein level as a PD diagnostic tool. *α*-Synuclein is a major component of the Lewy body, a pathological hallmark of PD, and overexpression of *α*-synuclein by gene duplication or triplication causes early onset PD in humans [14, 15]. All of these results indicate that the amount of *α*-synuclein is important in the pathogenesis and development of PD. Both enzyme-linked immunosorbent assay and immunoprecipitation analyses have been used to measure *α*-synuclein levels in plasma or CSF. However, these results varied and were occasionally contradictory [12], although an increase in oligomeric *α*-synuclein level in CSF of patients with PD has been reported [13, 33]. Because of the importance of *α*-synuclein as a PD marker protein, we also tested whether our urine exosome samples contain *α*-synuclein, despite previous reports not detecting *α*-synuclein in a proteomic analysis of urine exosomes [10, 11, 18]. Our Western analysis detected no monomeric *α*-synuclein band in the urine exosome samples except some samples with proteinuria or the positive control (Figure 2 lanes A-45, B-40, and D-SH). Paradoxically, detection of monomeric *α*-synuclein in some proteinuria samples supported our decision to exclude the proteinuria samples from analysis.

DJ-1 is an antioxidant protein that is autoxidized when exposed to oxidative stress and protects cellular contents and regulates the gene expression of antioxidative defense [34, 35]. Because oxidative stress is suspected as one of major causes of PD, DJ-1 and oxidized DJ-1 were extensively studied for their potentials as PD biomarkers using various biofluids such as CSF, blood, and saliva [35]. However, comparison of DJ-1 levels in CSF and blood between PD and non-PD cases were contradictory [36]. A recent study reported that DJ-1 level in saliva is higher in advanced stage of PD patients than non-PD controls [36]. Another study reported that the 4-hydroxy-2-nonenal modification of DJ-1 in whole blood cells is altered in late-stage PD [37]. Both studies suggested the potential for DJ-1 as a PD biomarker. It will be worth determining whether DJ-1 in PD urine exosomes contains a more oxidized form of DJ-1.

A gender difference in PD-related protein levels has been reported previously [24, 26, 38]. For example, Caranci et al. reported that plasma *α*-synuclein levels are lower in males than those in females with advanced PD [24]. Protein levels of LRRK2 and DJ-1 in our study clearly showed a gender difference (Figure 3). In particular, DJ-1 showed almost a threefold increase in females (*P* < 0.0001) regardless of disease status (Figure 3(e)). In contrast to DJ-1, LRRK2 levels were approximately twofold lower in females (*P* < 0.01) in our study regardless of disease status (Figure 3(b)). We do not know the exact reason for the gender differences of LRRK2 and DJ-1 at present. At least, estrogen might not be a cause, because all females in our study were postmenopausal. The gene expression patterns in normal and PD dopaminergic neurons were reported to be gender-specific [26, 38]. The lower amounts of LRRK2, the PD dominant gene product, and the higher amount of DJ-1, the PD recessive gene product, in females might be related to the observance that PD occurs in males at a much higher frequency than that in females [39], although it requires more study. Downregulation of DJ-1 was observed in male PD under stringent analysis, although it was also observed in both genders when relaxed condition was applied [26]. More research is needed to explain the gender differences of LRRK2 and DJ-1.

Overall, our analysis showed the possibility that DJ-1 could be used as a biomarker for PD diagnosis through urine exosome analysis, at least in males, although a well-controlled study with more samples is required to validate our hypothesis.

## 5. Conclusion

We compared levels of PD causative proteins such as LRRK2, *α*-synuclein, and DJ-1 in urine exosomes obtained from a Korean PD and non-PD population. The Western analysis showed the presence of only LRRK2 and DJ-1, but not *α*-synuclein. In addition, a significant difference in DJ-1 level was observed between the PD and the non-PD groups only in male samples whereas no difference in LRRK2 was observed between PD and control samples even when gender was considered. Although our sample group was too small to conclude the significance of this finding, this result is significant enough to warrant further analysis with a larger sample size.

## Figures and Tables

**Table 1:** Summary of clinical samples.

	Non-PD		PD^a^	
Gender	Male	Female	Male	Female
Number	10	11	14	12
Age (years)	70 ± 3.2	71 ± 3.2	73 ± 2.1	73 ± 2.6
Disease duration (years)	NA^b^	NA	5.8 ± 0.60	4.8 ± 0.60

^a^Patients' Parkinson's disease (PD)-related criteria such as Hoehn/Yahr or Unified Parkinson's Disease Rating Scale scores were unavailable.

^b^NA: not applicable.
